# Development and validation of a short-form (6-item) version of the clinician-administered dissociative states scale (CADSS-SF)

**DOI:** 10.1080/20008066.2026.2678662

**Published:** 2026-06-15

**Authors:** V. Ursule Taujanskaite, Sunjeev K. Kamboj

**Affiliations:** Clinical Psychopharmacology Unit, University College London, London, UK

**Keywords:** Dissociation, CADSS, nitrous oxide, ketamine, short-form scale, Disociación, óxido nitroso, ketamina, CADSS

## Abstract

**Background:** State dissociation is commonly assessed using the Clinician Administered Dissociative States Scale (CADSS; 19-items). A briefer CADSS would have many advantages, enabling assessment of transient dissociative states and allowing repeated assessment within short intervals while minimizing participant burden. This is especially relevant in studies of dissociative drugs which cause sedation and psychomotor slowing. Here we describe the process of developing a short-form version of the CADSS (CADSS-SF), based on nitrous oxide (N_2_O) - induced dissociative responses in healthy volunteers.

**Methods:** In the ‘development phase’, using data from three experimental pharmacological studies on N_2_O in healthy volunteers (*n* = 229), we identified the most ‘N_2_O-responsive’ items with the highest item-total correlations, endorsed by experts and consistent with published accounts on the phenomenology of drug-induced dissociation. Identified items were subjected to confirmatory factor analysis using a separate validation dataset (*n* = 80), which tested a series of one- and two-factor models with 6–8 items.

**Results:** A 6-item, single-factor CADSS-SF consisted of derealization and depersonalization items and showed excellent model fit (*χ*^2^(9) = 0.246, *p* = .246, CFI/TLI>0.99, RMSEA = 0.059). The CADSS-SF was internally consistent (w = 0.87), correlated strongly with the full scale (*r* ≥ 0.88) and moderately with a measure of the related, but distinct construct of psychotomimesis (*r* = 0.63).

**Discussion:** The CADSS-SF is a promising tool for rapid assessment of dissociation. It may be useful for capturing fleeting experimentally-induced dissociative phenomena that would otherwise be disrupted through the process of extended self-reporting, or for studying dissociation during drug intoxication, which is often accompanied by psychomotor slowing, sedation and inattention. The scale's brevity may allow tracking of changes in dissociation over relatively brief periods. However, like the parent long-form (19-item) CADSS, the CADSS-SF primarily captures variations in derealization/depersonalization only and may therefore be less appropriate for capturing the multidimensionality of dissociative phenomena. Further validation is required to establish the generalisability of the CADSS-SF beyond experimentally-(drug-) induced dissociation in healthy populations.

## HIGHLIGHTS

- Development of a short-form Clinician-Administered Dissociative States Scale (CADSS-SF), which minimizes participant burden and enables rapid assessment of drug-induced dissociation.
- Item selection was based on sensitivity to nitrous oxide and expert recommendation. Confirmatory factor analysis supported a unidimensional structure for CADSS-SF.
- CADSS-SF showed high internal consistency and strong correlation with the 19-item CADSS.

## Abstract (es)

**Antecedentes**: El estado de disociación suele evaluarse mediante la Escala de Estados Disociativos Administrada por el Clinico [*Clinician-Administered Dissociative States Scale* (CADSS; 19 ítems)]. Una versión más breve de la CADSS tendría múltiples ventajas, permitiendo evaluar estados disociativos relativamente transitorios y realizar evaluaciones repetidas en intervalos cortos, minimizando al mismo tiempo la carga para los participantes. Esto es especialmente relevante en estudios sobre fármacos disociativos, los cuales producen sedación y enlentecimiento psicomotor. En este trabajo describimos el proceso de desarrollo de una versión abreviada de la CADSS (CADSS-SF).

**Métodos:** En la ‘fase de desarrollo’, utilizando datos provenientes de tres estudios farmacológicos experimentales sobre óxido nitroso (N2O) en voluntarios sanos (*n *= 229), identificamos los ítems con mayor respuesta al N2O, con las correlaciones ítem-total más elevadas, respaldados por expertos y consistentes con descripciones publicadas sobre la fenomenología de la disociación inducida por fármacos. Los ítems inicialmente identificados fueron sometidos a análisis factorial confirmatorio utilizando un conjunto de datos de validación independiente (*n *= 80), en el cual se evaluó una serie de modelos unifactoriales y bifactoriales con 6 a 8 ítems.

**Resultados:** Una CADSS-SF unifactorial de 6 ítems mostró un excelente ajuste del modelo (χ²(9) = 0.246, *p* = .246, CFI/TLI>0.99, RMSEA = 0.059). La CADSS-SF fue internamente consistente (α = 0.87) fuertemente correlacionado con la escala completa (r≥0.88).

**Discusión:** La CADSS-SF constituye una herramienta prometedora para la evaluación rápida de la disociación. Puede ser especialmente útil para captar fenómenos disociativos transitorios inducidos experimentalmente que, de otro modo, podrían verse alterados por el propio proceso de autorreporte prolongado, o para estudiar la disociación durante estados de intoxicación por drogas, los cuales suelen acompañarse de enlentecimiento psicomotor, sedación e inatención. La brevedad de la escala podría permitir el seguimiento de cambios en la disociación a lo largo de períodos relativamente breves. Sin embargo, podría ser menos adecuada para captar la multidimensionalidad de los fenómenos disociativos.

## 1. Introduction

The dissociative anaesthetics ketamine and nitrous oxide (N_2_O) are rapidly acting antidepressants, with wide-ranging pharmacological effects (Breault et al., 2025). Like other classic and atypical psychedelic drugs, ketamine and N_2_O produce pronounced subjective changes during drug administration. Among the most salient of these is ‘dissociation’, which refers to a breakdown in the normal continuity and coherent integration of identity, memory, perception and behaviour (American Psychiatric Association, 2022). Dissociation is a transdiagnostic phenomenon and an established empirical and theoretical construct, predating Janet’s systematic description of dissociative phenomena in the nineteenth century (Hart & Dorahy, 2023). Descriptions of its theoretical basis, role in posttraumatic stress disorder (PTSD) and measurement can be found in many excellent recent reviews (e.g. Atchley & Bedford, 2021; Lynn et al., 2022; Wainipitapong et al., 2025).

In addition to PTSD, varying levels of dissociative symptoms are also seen in a range of other psychological disorders, including borderline personality disorder (BPD), and schizophrenia (Lyssenko et al., 2018). Dissociation is a core symptom in dissociative disorders and the dissociative subtype of PTSD, which accounts for 14.4% of PTSD cases (Stein et al., 2013). However, dissociation is also a normative state occurring on a (non-normally-distributed) continuum, with milder, subclinical levels being present in the general population (Maaranen et al., 2008; Ray & Faith, 1995; Ross et al., 1990).

Although the ‘ecological validity’ of experimentally-induced dissociation has not yet been fully investigated (Heekerens & Schmahl, 2025), some evidence suggests that drug-induced and naturally occurring pathological dissociation in trauma-exposed individuals has sufficient similarities as to allow the former (especially ketamine and N_2_O-induced dissociation) to serve as useful experimental models for pathological dissociation. Firstly, there are some striking parallels in the resting-state brain network dynamics observed in dissociative anaesthetic-induced dissociation on one hand, and in PTSD patients on the other (Goldway et al., 2026). Secondly, ketamine-induced (van Schalkwyk et al., 2018) and N_2_O-induced dissociation (Piazza et al., 2022) share psychometric similarities to dissociation in PTSD (Bremner et al., 2024), with measured items coalescing around one or two putative latent factors (derealization and depersonalization). Relatedly, the extent or ‘severity’ of acute ketamine-induced and N_2_O-induced dissociation is similar to background or script-driven dissociation levels seen in PTSD (Brake et al., 2025).

Beyond similarities to natural-type clinical phenomena, drug-induced changes in subjective states – including dissociation – are important clinical phenomena in their own right. For example, recovery from traumatic stress symptoms may be delayed when the traumatic event (specifically, sexual assault) occurs in the context of substance use, which might include ketamine, alcohol, cannabis, and other dissociation-inducing drugs (Gong et al., 2019). The clinical importance of drug-induced dissociation is also highlighted by the increased risk of traumatic stress symptoms when a dissociative anaesthetic (but not opioid analgesic) is administered shortly after a traumatic event (Schönenberg et al., 2005), suggesting that acute *post*-traumatic – as well as peritraumatic – dissociation may have a role in the aetiology of acute and posttraumatic stress disorder.

Generally considered an off-target effect of psychiatric treatments (e.g. esketamine for depression) that should be minimized (e.g. Pereira et al., 2021), dissociative subjective responses – including drug-induced dissociation – also appear to be informative clinical signals that may predict treatment outcome (e.g. Niciu et al., 2018). Moreover, in the case of naturally-occurring dissociation, early dissociative symptoms may be useful prognostic indicators for the development of PTSD (Briere et al., 2005; Lebois et al., 2022) and psychosis-like experiences (Perona-Garcelán et al., 2012; Varese et al., 2012). As such, drug-induced dissociation in healthy participants is a potentially valuable method for reversibly modelling psychopathological dissociative symptoms and offers a platform for investigating the neurobiological and cognitive-psychological processes that interact to produce this anomalous state.

Dissociation is commonly investigated as a stable individual difference (trait) or a transient state-level phenomenon (often in the context of behavioural or pharmacological manipulations). Both enduring and fleeting dissociation are typically characterized by detachment from the bodily self (depersonalization) and/or reality (derealization), and/or a perceived loss of control over volitional cognitive capabilities (e.g. amnesia), so-called ‘compartmentalization’. The presence of these descriptively distinct constructs is at least partially supported by psychometric evidence (Niciu et al., 2018; Piazza et al., 2022). On the other hand, the clinical phenomena of detachment (derealization and depersonalization) are more clearly aligned with the effects of dissociative anaesthetics than compartmentalization phenomena (Griffiths et al., 2021; Mollaahmetoglu et al., 2021; Sumner et al., 2021).

The most commonly used tool for assessing dissociative states, especially in clinical and experimental psychopharmacological studies (Short et al., 2018), is the Clinician-Administered Dissociative States Scale (CADSS; Bremner et al., 1998). The original version of the CADSS was devised to assess traumatic stress-related dissociation and was a combination of self – and clinician-rated items. The scale was subsequently adapted for use as an exclusively self-report 19-item measure. Further adaptations included expanding the item pool to broaden the coverage of dissociative phenomena (23-item version). Among studies of experimental induction of dissociation that reported version information, the 19-item CADSS was the most commonly used long-form version (Brake et al., 2025).

Although the long-form (19 or 23 item) CADSS has been validated in clinical samples with naturally occurring dissociation (Bremner et al., 2024), its validity may be compromised under certain experimental conditions. For example, in experiments investigating behaviourally induced dissociative states (e.g. mirror gazing), the very process of self-reporting might disrupt the fragile subjective state induced through the experimental manipulation. This may also be relevant for relatively short-lived dissociative states, e.g. during experimentally induced peritraumatic dissociation or during script-driven traumatic dissociative responding. Alternatively, while the continuous dissociative state produced by ketamine or N_2_O administration may be less likely to be disrupted through questionnaire completion, drug-induced cognitive deficits (especially inattention, psychomotor slowing, reduced general alertness; Oranje et al., 2000; Micallef et al., 2002; Fried et al., 1995) may affect the rate and reliability of responding relative to non-drugged states.

In the case of rapidly acting antidepressants such as ketamine and N_2_O, dissociation is but one of many relevant subjective states, any of which may prove to be an important marker of pro– or countertherapeutic effects (psychedelic-like effects, psychotomimesis, sedation, stimulation, general subjective drug sensitivity, such as ‘feel’ or ‘high’, and positively and negatively reinforcing drug effects). Given, therefore, that researchers may use multiple self-report measures to assess the range of drug-induced subjective states, participant burden may be compounded while they are substantially impaired. Considering also that the internal consistency of the long form CADSS is high (*α* > 0.9; Bremner et al., 1998, 2024), it is possible that there is some item redundancy. These considerations suggest that a briefer version of the CADSS may have advantages in some circumstances, especially where the full coverage of dissociative phenomena is less important. The current study therefore aimed to evaluate a short-form version of the CADSS for assessing drug-induced dissociative experiences. A shortened CADSS scale has been published based on dissociative experiences during subanaesthetic ketamine administration in people with treatment-resistant depression (Rodrigues et al., 2021). However, a substantial proportion of patients with depression report significant co-occurring dissociative symptoms (Fung et al., 2022); it is therefore possible that the scale developed by Rodrigues and colleagues was not based on the dissociative effects of ketamine alone, but rather the additive or interactive effects of a dissociative anaesthetic *and* psychopathological dissociative symptoms. The extent to which Rodrigues et al.’s (2021) brief CADSS is also applicable to ‘modelled’ or drug-induced dissociation in healthy participants is unknown. Similarly, it is not clear whether Rodrigues et al.’s (2021) brief CADSS has broader application to drugs other than ketamine.

N_2_O has many parallels with ketamine, including overlapping cellular and molecular mechanisms of action (Izumi et al., 2022). Their profile of effects in human psychopharmacological studies is similar (Piazza et al., 2022; Walsh et al., 2022) as is their therapeutic profile as rapidly-acting anti-depressants (Rech et al., 2024). These findings suggest that these drugs can substitute for one another. Given the growing interest in the therapeutic potential of dissociative anaesthetics as psychiatric treatments (Liu et al., 2022; Walsh et al., 2022), including for PTSD (Yin et al., 2026) and the value of these drugs as models of psychopathological dissociation, there is a continued need for a sensitive, yet brief, real-time measure of acute dissociation. Such a measure might have special utility under conditions of drug-induced impairment, when efforts should be made to reduce participant burden.

As such, the current study aimed to develop a brief version of the CADSS based on a pharmacological model of pathological dissociation. To realize this aim, we gathered data from multiple studies in which the CADSS long form was used to assess dissociation induced by a single dose of 50%-N_2_O in healthy volunteers. We aimed first to identify CADSS items that seemed particularly relevant and sensitive to the effects of N_2_O, while aiming to retain adequate coverage of the concept of dissociation (i.e. covering derealization *and* depersonalization). Thereafter, we sought to validate the resulting short form measure using confirmatory factor analyses. Based on related prior research with the long form CADSS, a single ‘general dissociation’ factor observed in PTSD patients (Bremner et al., 2024; NB this diverged from the originally proposed three-component conceptualization of CADSS as a measure of dissociation in PTSD; Bremner et al., 1998) was tested alongside a two-factor model suggested in our previous work evaluating the full scale CADSS in participants receiving 50%-N_2_O (Piazza et al., 2022).

## 2. Methods

### 2.1. Participants

Participant data was drawn from four separate studies (see *Datasets*). Inclusion criteria were age 18–50 years and good general health. Exclusion criteria included drug or alcohol dependence, use of illicit drugs >1/week, contraindications to N_2_O (collapsed lung or any other respiratory disorder, recent dental surgery, ear or nose infection or inflammation, vitamin B12 metabolism disorders, anaemia), history of any neurological or cardiovascular problems, diabetes, liver or kidney disorders, high or low blood pressure, pregnancy or current breastfeeding, and current use of psychiatric, glucocorticoid modulating, or cardio-active drugs.

Participants were recruited through local adverts and online platforms (e.g. Call for Participants). Upon responding to study advertisements, they were asked to complete a preliminary online screening, and if they met broad inclusion criteria, underwent a full eligibility screening telephone interview.

### 2.2. Procedure

All studies were carried out at the Clinical Psychopharmacology Unit at University College London (UCL), between 2016 and 2024, and all procedures were approved by the UCL Research Ethics Committee. All participants were generally healthy adult volunteers and provided written informed consent. The aims of these studies varied and are outlined below (see *Datasets*).

Additional procedural details are outlined in the relevant publications (Das et al., 2018; Kamboj et al., 2021; see also Piazza et al., 2022). Critically, these published studies, as well as the unpublished studies, were similar in terms of the key methodological details. Specifically, dissociation was assessed using the CADSS prior to, and during a single gas inhalation session (N_2_O or placebo, where relevant; see *Drug administration* below).

The long-form CADSS containing the same 19 items across studies was completed at pre- and peri-inhalation alongside a number of other questionnaires (see Kamboj et al., 2021), with responses recorded directly onto an online survey platform by the participant (Qualtrics, Provo, UT). N_2_O or placebo was administered for 30-40 min by trained research staff, and peri-inhalation questionnaires were completed after an equilibration period of ∼5 min.

### 2.3. Drug administration

Across all studies, participants either inhaled Entonox (50% N_2_O premixed with 50% oxygen, BOC, UK) or medical air (BOC, UK) via identical mouthpieces and demand valves. Cylinders were covered using opaque sleeves to conceal treatment condition from participants.

### 2.4. CADSS

The 19-item version ‘full-scale’ CADSS was used in all studies. This contains items intended to tap depersonalization (items 3, 4, 5, 6, and 7), derealization (items 1, 2, 8, 9, 10, 11, 12, 13, 16, 17, 18 and 19) and amnesia (items 14 and 15). Each item is rated on a scale from 0 (not at all) to 4 (extremely) and as such, the range of scores is 0–76 for the full scale; similarly, each subscale score is determined through summing items within that subscale. All items were used in the development phase, but only items selected for the short form were used in the validation phase.

The CADSS-19 was originally devised as a clinician-administered tool for assessing trauma-related dissociative states (Bremner et al., 1998) and was subsequently adapted for self-report. The scale has been validated in clinical samples (Bremner et al., 2024) but has also been commonly used in studies assessing experimentally induced dissociation (Brake et al., 2025). The CADSS has high internal consistency (*α* > 0.9; Bremner et al., 1998, 2024), and previous work has shown reasonable fit within one or two factor models (Bremner et al., 2024; Piazza et al., 2022).

### 2.5. Datasets

The development dataset (*n*_DEV_ = 229) consisted of participants receiving N_2_O (*n*_N2O(DEV)_ = 160) or placebo medical air (*n*_PBO(DEV)_ = 69) and was intended to determine the sensitivity of individual items of the CADSS using within-group (pre- versus peri-) and between-groups (N_2_O versus placebo) comparisons. The development dataset was drawn from three studies: Studies 1, 2 and 3 (Studies 2 and 3 were placebo controlled) that shared key methodological features. Participants completed the CADSS before and during N_2_O inhalation: Study 1 (Das et al., 2018): *n*_N2O(S1)_ = 60; Study 2 (Kamboj et al., 2021): *n*_N2O(S2)_ = 40; Study 3 (unpublished): *n*_N2O(S3)_ = 60 *or* before and during medical air (placebo) inhalation: Study 2 (Kamboj et al., 2021): *n*_PBO(S2)_ = 40; Study 3 (unpublished): *n*_PBO(S3)_ = 29. There was minimal missing data in the development dataset (a maximum of 0.87% for any single CADSS item across the sample), which was handled through listwise deletion.

The validation dataset (*n*_VALID_ = 120) was from a separate study: Study 4 (unpublished): *n*_N2O(S4)_ = 80, n_PBO(S4)_ = 40. The placebo data were only used to test the sensitivity of individual CADSS items to N_2_O versus placebo. All other analyses were performed only on participants receiving N_2_O in the development (*n*_N2O(DEV)_ = 160) or validation (*n*_N2O(VALID)_ = 80) phases. There was no missing data in the validation dataset.

Studies varied broadly in their aims and designs, although, as noted above, the critical within-group and between-group effects of gas inhalation on CADSS were examined in an identical manner. In terms of the aims of the component studies, one of the published studies examined the effects of N_2_O on retrieval-dependent modification of alcohol-related memories and had no placebo condition (Das et al., 2018). The other published study (Kamboj et al., 2021) examined the effects of background depression and impulsivity on sensitivity to the rewarding effects of N_2_O compared to placebo (note that these were not clinical participants). Of the two unpublished studies, one examined the effects of N_2_O on retrieval-dependent modification of emotional memories. The other characterized the behavioural and subjective psychotomimetic effects of N_2_O. The latter three involved randomization to a placebo or N_2_O group (1:1 or 1:2 ratio). Allocation to condition in the randomized experiments was achieved using a true random number generator (https://www.random.org/sequences/) to produce non-repeating sequences of integers corresponding to participant ID, which were then assigned to drug condition in a 1:1 or 1:2 ratio (see Kamboj et al., 2021 for further details).

#### 2.5.1. Item selection

Initial item selection (development phase) was based on considerations of sensitivity, reliability, expert opinion and a review of phenomenological (qualitative) reports of drug-induced dissociation.

### 2.6. Statistical analysis

#### 2.6.1. Sensitivity to N_2_O's effects

For each item we examined the differences between peri-inhalation N_2_O and peri-inhalation placebo scores (between-groups sensitivity), using Wilcoxon rank-sum tests, and calculated effect sizes using rank-biserial correlations. Item-level sensitivity was also tested using Wilcoxon signed-rank tests to compare pre- versus peri-N_2_O scores (within-group sensitivity), and effect sizes were calculated using paired rank-biserial correlations. Item-total correlations (Spearman’s rank correlation coefficients) were tested for each item for peri-inhalation scores. Non-parametric tests were used due to the ordinal nature of items of the CADSS and the preponderance of the items showing positive skew. For each of the three ‘families’ of tests (between-group sensitivity, within-group sensitivity and item-total correlation), Bonferroni correction was applied to control the family-wise error rate.

To ensure clinical and practical relevance, experts (*n* = 3) also reviewed the CADSS items and provided recommendations for retention based on their understanding of the subjective effects of this drug class. The experts consisted of two Professors of Psychopharmacology and an Associate Professor of Experimental Psychology, each from a different research-intensive university in the UK, with extensive experience in dissociation and its measurement in the context of clinical disorders (*n* = 1) or experimental and clinical psychopharmacology (*n* = 2). The experts evaluated items independently and were blind to each other’s evaluations. Other than providing recommendations for item retention, they had no other involvement in the study. For each objective index (between-groups sensitivity, within-group sensitivity and item-total correlation), CADSS items were sorted in descending order based on the corresponding effect size, and the top ten items were selected. As for expert endorsement, CADSS items were considered ‘endorsed’ if a minimum of two of three experts had recommended their retention. This yielded four partially overlapping sets of items (three sets of ten and one set of seven). Items that appeared in all, or three of the four sets were retained, while items appearing in only one or two sets were discarded.

#### 2.6.2. Validation of the short-form CADSS: confirmatory factor analysis

After initial item selection, the retained items (*k* = 8 items) were subjected to a confirmatory factor analysis (CFA) in the N_2_O group of the validation dataset (*n* = 80). CFA (as opposed to exploratory factor analysis) was appropriate given that our primary goal was not to discover the number of factors, since there was already evidence for either a one or two-factor model. Specifically, previous work on the CADSS-19 has shown acceptable fit with one- and two-factor models (Piazza et al., 2022); NB the three-factor model tested in Piazza et al., 2022 is not relevant here given its untenability with ≤8 items; (Bandalos, 2018).

Factor loadings were freely estimated; factor variances were fixed to 1 and residual covariances to 0. For the two-factor model, F1 and F2 were allowed to covary. Model fit was evaluated using the Chi-squared test, and according to the Comparative Fit Index (CFI), the Tucker Lewis Index (TLI), root mean square error of approximation (RMSEA) and standardized root mean square residual (SRMR). CFI and TLI values > .90 and > .95 were considered as indicative of acceptable and good fit. For SRMR, these values were ≤  .10 and ≤  .08 respectively, while RMSEA values of ≤  .06 were considered indicative of good fit (Hu & Bentler, 1998, 1999).

The final short-form CADSS (CADSS-SF) scale was then evaluated in the full dataset (development and validation datasets combined: *N* = 349, of which *n* = 240 inhaled N_2_O, and *n* = 109 inhaled medical air). Internal consistency was assessed at peri-inhalation using McDonald’s omega (ω), with values greater than .70 considered acceptable fit (Mokkink et al., 2018). Validity of the shortened scale was evaluated by correlating the CADSS-SF with the full scale CADSS during N_2_O inhalation. Corrected coefficients were calculated to account for spurious inflation in correlation due to shared items between the short-form and the full scale (Levy, 1967; see supplement for formula).

Construct validity of the CADSS-SF was tested by correlating CADSS-SF total scores with the total score from the Psychotomimetic States Inventory (PSI; Mason et al., 2008) using Pearson’s *r*. Our previous work (Piazza et al., 2022), as well as various theoretical (e.g. Morrison et al., 2003) and empirical evidence (Longden et al., 2020), supports the notion that psychosis-like experiences captured by the PSI and dissociation are conceptually related but nonetheless distinct.

Data were analysed using R statistical software (R Core Development Team, 2025, version 4.4.2). CFAs were fit using the *lavaan* package (Rosseel, 2012), with the Weighted Least Squares (WLSMV) estimator, which provides robust parameter estimates and standard errors in models with ordinal data (Beauducel & Herzberg, 2006).

## 3. Results

### 3.1. Development sample characteristics

Participant characteristics of the development and validation datasets are outlined in Table 1. The placebo (medical air) group is only relevant to the development/item selection phase and was used to test between-groups item sensitivity. As shown in Table 1, participants in the two drug conditions in the development dataset had similar characteristics. Participants receiving N_2_O in the development and validation datasets were also similar.

**Table 1.** Participant characteristics for the development and validation phases.

	Development data set			
Drug condition	Placebo (*n* = 69)		N_2_O (*n* = 160)	
Female Male	36 (52%) 33 (48%)		77 (48%) 83 (52%)	
Age (SD)	25.7 (5.2)		25.3 (6.6)	
Educational level				
Highschool (secondary)	13 (19%)		34 (21%)	
Undergraduate	38 (55%)		88 (55%)	
Postgraduate	18 (26%)		38 (24%)	
Dissociation	Pre-inhalation	Peri-inhalation	Pre-inhalation	Peri-inhalation
CADSS (19-item total)	1.67 (2.96)	2.87 (5.19)	2.38 (4.53)	15.74 (13.60)
	Validation data set^1^			
	Placebo (*n* = 40)		N_2_O (*n* = 80)	
Female Male	30 (75%) 10 (25%)		60 (75%) 20 (25%)	
Age (SD)	24.8 (5.2)		24.8 (5.1)	
Educational level				
Highschool (secondary)	6 (15%)		6 (8%)	
Undergraduate	18 (45%)		49 (61%)	
Postgraduate	16 (40%)		25 (31%)	
Dissociation	Pre-inhalation	Peri-inhalation	Pre-inhalation	Peri-inhalation
CADSS (19-item total)	2.15 (2.75)	1.98 (2.56)	2.13 (2.73)	14.33 (15.87)

^1^ NB: the CFA only used the N_2_O data (*n* = 80)

### 3.2. Development of the short-form CADSS (CADSS-SF)

Preliminary item selection was based on group-level differences, sensitivity to N_2_O, and correlations with the total scale. Based on the effect sizes of each of these indices, together with expert recommendations for item retention and phenomenological reports of dissociative experiences (Griffiths et al., 2021; Mollaahmetoglu et al., 2021; Pomarol-Clotet et al., 2006; Sumner et al., 2021), eight items were retained for validation, listed in Table 2. A parallel table describing the relevant effect sizes and expert opinion for items that were not retained is presented in the Supplement (Table S1).

**Table 2.** Preliminary item selection in the development dataset (*n* = 229) based on effect sizes (i.e. item sensitivity of N_2_O v placebo, pre-N_2_O v peri-N_2_O and item-total correlation) and expert endorsement.

Item type (subscale) on original scale^1^	Item number on original scale	Item	Placebo-N_2_O difference (*r_rb_*)	Pre-peri N_2_O difference (*r_rb_*)	Item-total correlation (ρ)	Expert endorsed^2^
Derealization	1	Do things seem to be moving in slow motion?	0.461***	0.784***	0.585***	Yes
Derealization	2	Do things seem unreal to you, as if you are in a dream?	0.492***	0.761***	0.702***	Yes
Depersonalization	3	Do you have some experience that separates you from what is happening; for instance, do you feel as if you are in a movie or a play, or as if you are a robot?	0.403***	0.586***	0.679***	No
Depersonalization	6	Do you feel disconnected from your own body?	0.367***	0.695***	0.629***	Yes
Depersonalization	7	Does your sense of your own body feel changed; for instance, does your own body feel unusually large or unusually small?	0.444***	0.714***	0.647***	No
Derealization	9	Do objects look different than you would expect?	0.307***	0.607***	0.631***	No
Derealization	12	Does this questionnaire seem to be taking much longer than you would have expected?	0.369***	0.583***	0.654***	No
Derealization	16	Have sounds almost disappeared or become much stronger than you would have expected?	0.409***	0.677***	0.639***	No

Note: For effect sizes, *** indicates *p* < .001 (family-wise error controlled using Bonferroni adjustment).

^1^ An additional item that showed moderate-large effects on indices of within – and between-group sensitivity and item-total correlations was not retained. Unlike the other listed items, the phenomenon captured by the rejected item (item 15) was conceptualized as ‘compartmentalization-like’ (i.e. an ‘amnesia’ item) rather than depersonalization or derealization.

^2^ Yes: ≥2 experts endorsed item.

### 3.3. Validation of the CADSS-SF

We compared the fit of a series of one and two-factor models to establish the best fitting structure for the eight-item pool (Table 3).

**Table 3.** Model specification and fit statistics for models tested with CFA in the validation dataset (N_2_O-group only, *n* = 80).

	Factors	Items	χ²	*df*	*p*	CFI	TLI	SRMR	RMSEA (90% CI)	F1 ∼∼ F2
1	Two	F1: 1, 2, 9, 12, 16&&F2: 3, 6, 7	47.291	19	< .001	.968	.953	.084	.137 (.089, .187)	.934
2	One	F1: 1, 2, 3, 6, 7, 9, 12, 16	49.977	20	< .001	.966	.953	.086	.138 (.090, .186)	–
3	Two	F1: 1, 2, 9, 16&&F2: 3, 6, 7	23.106	13	.040	.988	.981	.060	.099 (.021, .164)	.927
4	One	F1: 1, 2, 3, 6, 7, 9, 16	26.757	14	.021	.985	.978	.064	.107 (.041, .169)	
5	Two	F1: 2, 9, 16&&F2: 3, 6, 7	11.019	8	.201	.996	.992	.046	.069 (.000, .159)	.974
6	One	F1: 2, 3, 6, 7, 9, 16	11.452	9	.246	.997	.994	.048	.059 (.000, .147)	–

The *χ*^2^ (and associated *p* values) and RMSEA values of models 1 and 2 suggested model misspecification. Considering item 12 had the lowest factor loadings of all items in both models (λ ≈ 0.55) and the fact that it was not endorsed by any of our experts, led us to test models 3 and 4, which excluded item 12. These models showed a substantial reduction in *χ*^2^, although *p*-values were still <0.05 and RMSEA values continued to suggest inadequate model fit.

**Figure 1.**
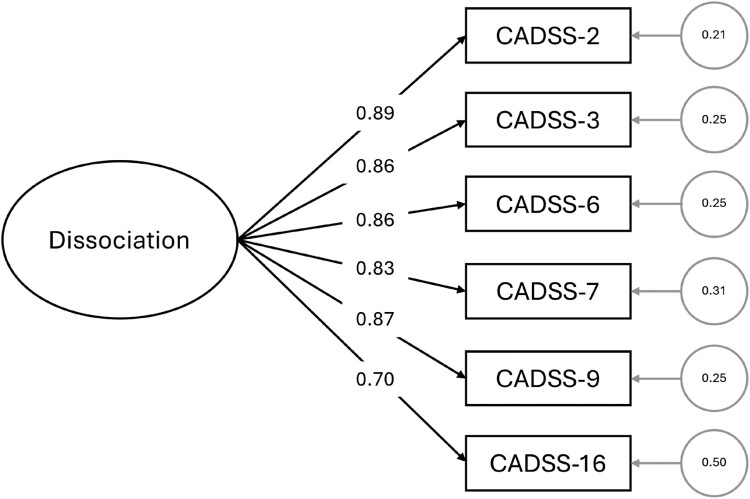
Path diagram of the one-factor solution for the 6-item CADSS pool (model 6). Coefficients on black arrows represent standardized factor loadings, grey circles represent residual variances.

Assessment of modification indices suggested that meaningful improvement in fit would be achieved by allowing the residuals of items 1 and 2 to covary. The two items were moderately strongly correlated (*r* = .716), suggesting potential redundancy. The inclusion of a correlation between their residuals would imply that they share item-specific variance not accounted for by the latent factor(s). We therefore excluded item 1 (which had lower factor loadings compared to item 2) and tested the fit of the 6-item models with one and two factors (models 5 and 6, Table 3). All of the fit indices suggested at least reasonable model fit using items 2, 3, 6, 7, 9 and 16. Factor loadings for the resultant 6-item (one-factor) model are shown in Figure 1. The adequacy of our sample size (*n* = 80) for reliable recovery of parameter estimates was evaluated in a simulation study (reported in the Supplementary Materials).

To further test its construct validity, CADSS-SF total scores were correlated with total scores on the Psychomimetic States Inventory (PSI), both measured peri-N_2_O in the validation sample. The CADSS-SF and PSI scales were moderately correlated, *r*(78) *=* 0.632, *p* < .001, confirming the expected association between these related but distinct constructs. The expected association was similarly strong between the long form CADSS and PSI in the current sample, *r*(78) *=* 0.634, *p* < .001.

### 3.4. Internal consistency of the CADSS-SF and correlation with long-form CADSS

The CADSS-SF showed good internal consistency, with ω = 0.87 (*n* = 240; combined development and validation datasets; single factor). The peri-inhalation total of the short-form also showed virtually perfect agreement with the full scale (*r* = 0.933). To account for inflation due to shared items, a more conservative estimate was obtained by calculating the corrected coefficient (Levy, 1967; see supplement for formula), yielding a correlation of *r* = 0.826. These values (*r* = 0.826–0.933) indicate good convergence with the long-form CADSS.

Using the full sample (i.e. combined development and validation samples, *n* = 349; *n*_N2O_ = 240; *n*_PBO_  = 109), the CADSS-SF also showed high between-groups sensitivity (peri-inhalation N_2_O v placebo; *V* = 3560, *p* < .001, *r_rb _= *0.593) and within-group sensitivity to the effects of N_2_O (pre v peri change in the N_2_O group; *V* = 189, *p* < .001, *r_rb _= *0.831).

## 4. Discussion

In this study, we developed and validated a short-form version of the CADSS (CADSS-SF). In addition to considerations of individual item sensitivity, we aimed for adequate representation of key dissociative phenomena likely to be important in studies of dissociative drugs like N_2_O and ketamine. As such, we integrated expert recommendations and published phenomenological reports in our decision-making during initial item selection.

The resultant 6-item CADSS-SF showed strong correlation with the original long-form (19-item) scale, suggesting substantial overlap in the construct measured by the two forms. CADSS-SF was highly sensitive to drug effects and provided clear distinction between the placebo and N_2_O (between-groups sensitivity), as well as between pre – and peri-inhalation in the N_2_O group (within-group sensitivity). The CFA model in which we loaded all 6 items onto a single latent factor also showed excellent fit statistics across the range of fit indices. Together, these findings provide preliminary support for the idea that the CADSS-SF is a valid short-form measure of acute drug-induced dissociation.

It is worth noting that another 6-item CADSS (the ‘simplified CADSS’) has been proposed (Rodrigues et al., 2021). While there is overlap between Rodrigues et al.’s (2021) simplified CADSS and our CADSS-SF (items 2, 6 and 7 are present in both), there are also important differences. Firstly, the simplified CADSS was designed to assess dissociative symptoms elicited by ketamine administration in patients diagnosed with major depression. Given that dissociation is part of the range of symptoms reported in depression (Fung et al., 2022), the results reported by Rodrigues et al. (2021) represented an evaluation of ‘acute on chronic’ dissociation. Furthermore, the development of the simplified CADSS used the 23-item CADSS as the starting point, consisting of the 19-item version plus four items referring to identity fragmentation and dissociative amnesia – aspects that are mostly absent from the 19-item version.

As noted above, although drug-induced cognitive impairment (including amnesia) may be a feature of dissociative drugs, the amnesia items in the long-form CADSS may not be as representative of drug-induced dissociative phenomena as the depersonalization and derealization items. Indeed, responses to such items may be difficult to distinguish from responses reflecting perceived drug-induced memory impairment. Given these considerations, and the fact that perusal of qualitative accounts of dissociative drugs (e.g. Griffiths et al., 2021; Pomarol-Clotet et al., 2006; Valtonen et al., 2024) (as well as our own extensive experience of the experimental psychopharmacology of these drugs) does not suggest that dissociative amnesia is a prominent feature of these drugs, the single potential amnesia item (item 15; see footnote to Table 2) that could have been included in the CADSS-SF was rejected. In contrast, Rodrigues et al.’s ‘simplified-CADSS’ contains 2 amnesia items. Additionally, Rodrigues et al. (2021) used data pooled from ketamine studies, whereas our evaluation of the CADSS-SF was based on data from studies of N_2_O. However, given the substantial phenomenological (Piazza et al., 2022), clinical (Kalmoe et al., 2020) and pharmacological (Izumi et al., 2022) overlap between N_2_O and ketamine, we do not believe that this difference is an important source of divergence between our study findings. Finally, since the simplified CADSS (Rodrigues et al., 2021) has not been assessed using CFA, its validity is yet untested. Given that the simplified CADSS includes a dissociative amnesia item (‘Do you have gaps in your memory?’) present only in the 23-item CADSS (and not the 19-item version used as our starting point for the CADSS-SF), we were unfortunately unable to compare its performance to the CADSS-SF.

### 4.1. Limitations and considerations for future research

Psychometricians have outlined the risks to validity when the number of items on a validated scale is reduced (Smith et al., 2000). In particular, content validity is adversely affected because fewer facets of the target construct are represented and multidimensionality of the construct may not be fully recognized in an abbreviated scale. Related to this, any content validity-related limitations of the long-form scale upon which a brief scale is based are necessarily transmitted to the brief scale. In the case of the CADSS-SF, as noted above, one potential amnesia item (of only two in the 19-item version of the CADSS) was not included in the final six items, partly because the wording of the relevant amnesia item did not allow (nor was it designed or intended to allow) for drug-induced cognitive/memory impairment to be distinguished from dissociative amnesia.

In any case, the main aim of developing the CADSS-SF was to prioritize brevity while achieving *sufficient* (rather than full) phenomenological coverage of the most relevant aspects of drug-induced dissociation. Our impression from observing ∼500 participants from our studies of dissociative anaesthetics (ketamine and N_2_O; informal observations) suggested that issues relating to attentional drift, perseveration and sedation during ketamine or N_2_O intoxication are a greater risk to the validity of self-report measures than insufficient assessment of all facets of constructs of interest. In studies where comprehensive assessment is more critical (e.g. clinical trials involving patients with dissociative disorders or PTSD, etc.), the CADSS-SF may not be appropriate, and the full-length measure should be used.

Our two-stage approach, which involved initial item-level scrutiny and selection (development phase), could be criticized on psychometric groups given that it does not take into account the correlation between items, which reflect (an) underlying latent construct(s). Our approach, however, viewed responsiveness to experimental manipulation as central to our goal of developing a state-sensitive, maximally responsive measure rather than primarily being concerned with a structurally ‘clean’ latent variable scale. As such, we acknowledge that an alternative approach might have been to use an initial CFA with all available peri-N_2_O data as our starting point in item selection using factor loadings to guide decision-making. Related to this is the sample size in the validation study (*n* = 80). Although this might be at the lower end of an acceptable sample size in structural equation modelling (Bentler & Chou, 1987), our simulation study suggested adequate recovery and stable estimates of parameters with *n* = 80. It is nonetheless possible that our validation study was underpowered to detect model misfit.

The present sample was relatively homogeneous, consisting of healthy volunteers within a relatively narrow (young) age range. The only dissociation-inducing manipulation was N_2_O administration. We are therefore not able to comment on the measurement invariance of the CADSS-SF across important demographic factors such as age, clinical status or other drugs. Some studies suggest that dissociative experiences tend to decrease with age (Ross et al., 1990; Torem et al., 1992), but whether this decline also applies to sensitivity to drug-induced dissociation is unknown.

Furthermore, given the ongoing investigations into the therapeutic potential of N_2_O in depression and PTSD (Liu et al., 2022), it will be particularly important that the performance of CADSS-SF is tested in these clinical samples where the acute drug effects may partially overlap with disorder symptomatology (Fung et al., 2025; Fung & Cheung, 2024). Such clinical application of the CADSS-SF rests on the assumption that measures developed for one purpose (e.g. examining chronic, trauma-related dissociative adaptations) can be applied to another (assessing acute, experimentally-induced and reversible dissociative states). In the introduction, we outline the evidence for overlap in phenomenology, psychometrics and underlying neurobiology between dissociative anaesthetic-induced and pathological dissociation. Nonetheless, whether ketamine/N_2_O-induced dissociation is in fact an adequate clinical model requires direct comparisons of the two forms of dissociation in future studies.

Given the phenomenological parallels between N_2_O and ketamine (Piazza et al., 2022), it might be assumed that measurement invariance would be observed with ketamine. This should nonetheless be established, especially if the CADSS-SF is used in clinical trials of ketamine. Invariance across timepoints in longitudinal studies, as well as across other drugs with dissociative properties (e.g. cannabis and alcohol), should also not be assumed. Finally, further validation of the CADSS-SF is required (e.g. establishing rest-retest reliability and convergent and discriminative validity).

## Supplementary Material

Supplemental Material
