# Supplementary material for: Development and validation of a short-form (6-item) version of the clinician-administered dissociative states scale (CADSS-SF)
Source: Eur J Psychotraumatol. 2026 Jun 15;17(1):2678662. doi: 10.1080/20008066.2026.2678662 (PMC13270868; doi:10.1080/20008066.2026.2678662)
Supplement: Supplemental Material [file ZEPT_A_2678662_SM3799.docx]

**SUPPLEMENT**

Formula for corrected correlation

Levy (1967) proposed a correction of the correlation between long and short-form to account for inflation due to item overlap:

$${r'}_{tx}= r_{tx}-(1-r_{xx})\frac{\sigma_{x}}{\sigma_{t}}$$

Where:

- $r_{tx}$ is the observed correlation between the long-form (*t*) and the subset (short-form, *x*)
- $r_{xx}$ is the reliability of the short-form (ω)
- $\sigma_{t}$ and $\sigma_{x}$ are the standard deviations of the long and short forms
- ${r'}_{tx}$ is the corrected correlation between short and long form

CFA sample size: A simulation study

Early sample size recommendations for CFAs suggest a ratio of 5-10 observations per free parameter (Bentler & Chou, 1987), although the required sample size is recognised to ultimately depend upon model complexity, factor loading strength and missing data amongst other considerations (Jackson, 2001; Wolf et al., 2013). Our final CFA model (model 6) was relatively simple, consisting of a single latent factor with six indicators and 12 free parameters, resulting in an observations-to-parameter ratio of 6.7:1. To further evaluate estimation quality for this model, a Monte Carlo simulation study with 10,000 replications was conducted. The simulation showed full convergence in all replications, with no improper solutions or Heywood cases. Minimal relative bias (-0.010 to - 0.005) was observed for factor loadings, while residual variances showed small negative bias across replications (-0.032 to -0.026). Parameter estimates displayed moderate sampling variability (SD = 0.14 – 0.15 for loadings; SD = 0.18-0.20 for residual variances). Empirical 95% confidence interval coverage was close to the nominal level for factor loadings (0.940-0.944) and slightly lower for residual variances (0.912-0.922). Overall, these results indicate adequate parameter recovery and reasonably stable estimation under the specified model and sample size conditions.

| **Item type (subscale) on original scale** | **Item number on original scale** | **Item** | **Placebo-N_2_O difference**  **(*r_rb_*)** | **Pre-peri N_2_O difference**  **(*r_rb_*)** | **Item-total correlation**  **(ρ)** | **Expert endorsed^2^** |
| --- | --- | --- | --- | --- | --- | --- |
| Depersonalization | 4 | Do you feel as if you are looking at things from outside of your body? | 0.215* | 0.489*** | 0.690*** | Yes |
| Depersonalization | 5 | Do you feel as if you are watching the situation as an observer or a spectator? | 0.242** | 0.513*** | 0.620*** | Yes |
| Derealization | 8 | Do people seem motionless, dear, or mechanical? | 0.242** | 0.485*** | 0.620*** | No |
| Derealization | 10 | Do colours seem to be diminished in intensity? | 0.257** | 0.427*** | 0.450*** | No |
| Derealization | 11 | Do you see things as if you were in a tunnel, or looking through a wide angle photographic lense? | 0.226* | 0.530*** | 0.590*** | No |
| Derealization | 13 | Do things seem to be happening very quickly, as if there is a lifetime in a moment? | 0.224* | 0.424*** | 0.540*** | Yes |
| Amnesia | 14 | Have there been things which have happened during this questionnaire that now you can’t account for? | 0.246** | 0.422*** | 0.630*** | No |
| Amnesia | 15 | Have you spaced out, or in some way lost track of what was going on during this experience? | 0.400*** | 0.694*** | 0.680*** | Yes |
| Derealization | 17 | Do things seem to be very real, as if there is a special sense of clarity? | 0.262** | 0.367*** | 0.540*** | No |
| Derealization | 18 | Does it seem as if you are looking at the world through a fog, so that people and objects appear far away or unclear? | 0.346*** | 0.656*** | 0.600*** | No |
| Derealization | 19 | Do colours seem much brighter than you would have expected? | 0.239** | 0.549*** | 0.520*** | No |

**Table S1**. Items excluded at preliminary selection in the development dataset (n = 229) based on effect sizes (i.e. item sensitivity of N_2_O v placebo, pre-N_2_O v peri-N_2_O and item-total correlation) and expert endorsement. For effect sizes, *** indicates *p* < .001, ** - *p* < .01, * - *p* < .05 (corrected for multiple comparisons using Bonferroni adjustment).

| Item_1_ | Model 1 | | | | Model 2 | | | Model 3 | | | | Model 4 | | | Model 5 | | | | Model 6 | | |
| --- | --- | --- | --- | --- | --- | --- | --- | --- | --- | --- | --- | --- | --- | --- | --- | --- | --- | --- | --- | --- | --- |
|  |  | *λ* | *SE* | *R^2^* | *λ* | *SE* | *R^2^* |  | *λ* | *SE* | *R^2^* | *λ* | *SE* | *R^2^* |  | *λ* | *SE* | *R^2^* | *λ* | *SE* | *R^2^* |
| 1 (DR) | F1 | 0.73 | 0.06 | 0.54 | 0.73 | 0.06 | 0.53 | F1 | 0.75 | 0.06 | 0.56 | 0.75 | 0.06 | 0.56 | -- | -- | -- | -- | -- | -- | -- |
| 2 (DR) | F1 | 0.95 | 0.04 | 0.90 | 0.93 | 0.03 | 0.87 | F1 | 0.96 | 0.04 | 0.92 | 0.93 | 0.03 | 0.87 | F1 | 0.90 | 0.05 | 0.81 | 0.89 | 0.04 | 0.79 |
| 3 (DP) | F2 | 0.87 | 0.04 | 0.76 | 0.85 | 0.04 | 0.72 | F2 | 0.88 | 0.04 | 0.78 | 0.86 | 0.04 | 0.73 | F2 | 0.87 | 0.05 | 0.76 | 0.87 | 0.05 | 0.75 |
| 6 (DP) | F2 | 0.87 | 0.04 | 0.75 | 0.84 | 0.04 | 0.71 | F2 | 0.87 | 0.04 | 0.76 | 0.85 | 0.04 | 0.71 | F2 | 0.87 | 0.04 | 0.76 | 0.86 | 0.04 | 0.75 |
| 7 (DP) | F2 | 0.84 | 0.05 | 0.70 | 0.83 | 0.05 | 0.68 | F2 | 0.83 | 0.05 | 0.68 | 0.81 | 0.05 | 0.66 | F2 | 0.83 | 0.05 | 0.70 | 0.83 | 0.05 | 0.69 |
| 9 (DR) | F1 | 0.89 | 0.06 | 0.78 | 0.87 | 0.05 | 0.75 | F1 | 0.88 | 0.06 | 0.78 | 0.86 | 0.05 | 0.75 | F1 | 0.88 | 0.06 | 0.77 | 0.87 | 0.05 | 0.75 |
| 12 (DR) | F1 | 0.56 | 0.08 | 0.31 | 0.55 | 0.08 | 0.30 | -- | -- | -- | -- | -- | -- | -- | -- | -- | -- | -- | -- | -- | -- |
| 16 (DR) | F1 | 0.73 | 0.07 | 0.54 | 0.73 | 0.07 | 0.53 | F1 | 0.70 | 0.07 | 0.49 | 0.69 | 0.07 | 0.48 | F1 | 0.71 | 0.07 | 0.50 | 0.71 | 0.07 | 0.50 |

**Table S2.** Standardized factor loadings, standard errors and R^2^ for each of the items included in the six tested models.

^1^Where DR = Derealization subscale and DP = Depersonalization subscale in the original specification (Bremner et al., 199
